# The Interplay of WNT and PPARγ Signaling in Vascular Calcification

**DOI:** 10.3390/cells9122658

**Published:** 2020-12-10

**Authors:** Stefan Reinhold, W. Matthijs Blankesteijn, Sébastien Foulquier

**Affiliations:** 1 Department of Pharmacology and Toxicology, Cardiovascular Research Institute (CARIM), Maastricht University, 6200 MD Maastricht, The Netherlands; stefan.reinhold@maastrichtuniversity.nl (S.R.); wm.blankesteijn@maastrichtuniversity.nl (W.M.B.); 2 Department of Neurology, School of Mental Health and Neuroscience, Maastricht University, 6200 MD Maastricht, The Netherlands

**Keywords:** vascular calcification, inflammation, vascular smooth muscle cells, macrophages, atherosclerosis, cardiovascular disease, PPARγ, WNT, β-catenin

## Abstract

Vascular calcification (VC), the ectopic deposition of calcium phosphate crystals in the vessel wall, is one of the primary contributors to cardiovascular death. The pathology of VC is determined by vascular topography, pre-existing diseases, and our genetic heritage. VC evolves from inflammation, mediated by macrophages, and from the osteochondrogenic transition of vascular smooth muscle cells (VSMC) in the atherosclerotic plaque. This pathologic transition partly resembles endochondral ossification, involving the chronologically ordered activation of the β-catenin-independent and -dependent Wingless and Int-1 (WNT) pathways and the termination of peroxisome proliferator-activated receptor γ (PPARγ) signal transduction. Several atherosclerotic plaque studies confirmed the differential activity of PPARγ and the WNT signaling pathways in VC. Notably, the actively regulated β-catenin-dependent and -independent WNT signals increase the osteochondrogenic transformation of VSMC through the up-regulation of the osteochondrogenic transcription factors SRY-box transcription factor 9 (SOX9) and runt-related transcription factor 2 (RUNX2). In addition, we have reported studies showing that WNT signaling pathways may be antagonized by PPARγ activation via the expression of different families of WNT inhibitors and through its direct interaction with β-catenin. In this review, we summarize the existing knowledge on WNT and PPARγ signaling and their interplay during the osteochondrogenic differentiation of VSMC in VC. Finally, we discuss knowledge gaps on this interplay and its possible clinical impact.

## 1. Introduction

Cardiovascular disease (CVD) affects more than 420 million people worldwide, causing 18 million deaths annually. A recently published study postulated an increase to 23 million deaths by 2030, which will increase the global economic burden of CVD to USD 1.044 billion. About 70% of these deaths are due to coronary heart disease (CHD) or ischemic strokes, outcomes that are mainly caused by the vascular disease atherosclerosis [1,2,3].

The atherosclerotic plaque manifests as single or multiple pathological deposits in the aorta’s vascular wall or in its branching arteries. These deposits mainly consist of living and dead cells, as well as lipids that accumulate in the vessel wall. Ultimately, the plaque may narrow the affected artery, which induces ischemia in the supplied organs. This narrowing results in CVDs such as CHD, peripheral artery disease, as well as acute vascular events like myocardial infarction and stroke. Notably, patients with atherosclerosis frequently suffer from vascular calcifications (VC), an ectopic deposition of calcium phosphate crystals in the atherosclerotic plaque [2]. 

Atherosclerosis of the coronary arteries often leads to acute and chronic complications associated with coronary artery calcification (CAC). The prevalence of CAC alternates between 50 and 70% and depends on ethnicity, which was confirmed by the Multi-Ethnic Study of Atherosclerosis that compared the prevalence of CAC in the Black, White, Chinese and Hispanic population, finding the highest risk for CAC in the White population [4]. Moreover, elevated CAC increases the risk of coronary events such as myocardial infarction by 10-fold, whereas overall VC increases mortality by up to 4-fold [5,6]. Consequently, understanding risk factors for VC, such as oxidative stress, inflammation and abnormal mineral metabolism (hyperphosphatemia, hypercalcemia), is essential to develop new treatments or even to prevent VC. 

Atherosclerotic risk factors include metabolic diseases like type 1 and type 2 diabetes (T1D/T2D), chronic kidney disease (CKD) as well as lifestyle factors like Western diet, smoking and obesity [2]. The steady increase of metabolic diseases in emerging countries, due to the raising life expectancy combined with the introduction of the Western diet, is alarming. Many scientists expect this trend to continue, generating higher mortality rates, posing an enormous burden for global healthcare systems [1,7]. Lately, a study of the indigenous Tsimane population, leading a pre-industrial lifestyle, found that 85% of the population was free from CAC, highlighting the effect of diet and physical activity on VC [8]. In contrast, athletes with a high physical activity display a higher prevalence of CAC than sedentary people, while still having a lower incidence of CVD and longer life expectancy [9]. These varying effects of CAC in different population groups indicate that a change in diet and physical exercise may positively change the impact of VC. Consequently, it appears necessary to examine calcification morphology (see Section 1.1 of this review) to explain the differential effects of VC for vascular events.

This review aims to present a brief overview of VC pathology and the participating cells. At first, we will explain the similarities between VC and endochondral ossification, as well as the contributing role of inflammation. Subsequently, the respective roles of Wingless/Int-1 (WNT)- and Peroxisome proliferator-activated receptor γ (PPARγ) signaling in atherosclerosis and VC will be explained before discussing existing knowledge about their interplay as well as its relevance for VC.

### 1.1. The Pathology of Vascular Calcification

Historically, VC was assumed to be a passive consequence of vascular injury. However, research demonstrated that VC is an actively regulated process that involves inflammation, oxidative stress and mechanisms similar to endochondral ossification [10,11]. The loss of physiological calcification inhibitors such as Fetuin-A, Osteoprotegerin (OPG) or matrix GLA protein significantly contributes to VC. Nonetheless, a detailed discussion of this topic is beyond the scope of this review [12,13].

The presence and morphology of VC is a significant feature of an atherosclerotic lesion and seems essential to identify unstable plaques, which could help to predict acute vascular events [14]. Calcifications consist of calcium phosphate crystal deposits that can be distinguished by their location, in arterial intimal (AIC) and arterial medial calcification (AMC) as well as by their size for macro- (>15 µm) and microcalcifications (0.5–15 µm) [10]. Additionally, to its occurrence in atherosclerosis, AMC manifests as Monckeberg’s arteriosclerosis. AIC, on the other hand, only occurs in atherosclerotic plaques and is strongly associated with inflammation [10]. Both conditions seem to be mainly driven by the osteochondrogenic transition of vascular cells.

Microcalcifications, presumably, can coalesce to larger macrocalcifications, and more extensive calcifications can increase vascular stiffness, an important and independent predictor of CVDs [15,16]. In contrast, there is evidence that macrocalcifications with a sheet-like morphology stabilize the atherosclerotic plaque. Consequently, macrocalcifications could be predominantly beneficial by preventing plaque ruptures and, thus, vascular events [10,14]. 

The Tampere vascular study found that the gene expression in atherosclerotic plaques differs by up to 50% between arterial beds, including genes responsible for inflammation, extracellular matrix modulation and lipid uptake [17]. This genetic difference is the first stepstone to explain why 80% of the plaques found in the peripheral arteries of the lower limbs show extensive, sheet-like calcifications, whereas microcalcifications occur in 43% and 47% of the plaques from the coronary arteries and the abdominal aorta, respectively [18]. Many studies demonstrated that microcalcifications can enhance the mechanical and hemodynamic stress on the fibrous cap of the plaque and, thus, increasing the chance of plaque ruptures [10,14]. This suggests that plaque ruptures occur more often in the thoracic aorta and the coronary arteries than in the lower-limb arteries. Besides the vascular topography, the embryonic origin of the contributing cells, and the etiology of the preexisting disease contribute to the heterogeneous calcifying properties of the atherosclerotic lesion [18,19,20]. 

Various, commonly used drugs and their effects on VC were studied in the last decades, including Statins, PCSK-9 inhibitors, calcium channel blockers, bisphosphonates and Renin-Angiotensin System (RAS) inhibitors [21,22]. Statins and the novel PCSK9-Inhibitors are used to lower the lipid levels in patients, and because of their anti-inflammatory effects, they were suggested to be beneficial for treating VC. Clinical studies for both groups showed conflicting results, mostly demonstrating a plaque stabilizing and pro-calcifying effect [21,22,23]. Similar, discouraging results regarding VC were found for antihypertensive RAS Inhibitors, bisphosphonate, and for calcium channel blockers [22]. Despite the limited number of studies, a promising option for the treatment of VC could be Vitamin K, essential for the carboxylation of the calcification inhibitor matrix GLA protein, which decreases during the progression of CKD [12,22]. Taken together, there are many different approaches and several new drugs are studied to tackle VC. However, there is no evidence that the mentioned drugs directly affect the detrimental, osteochondrogenic differentiation of VSMC. 

While treating VC seems complex, its proper diagnosis is also puzzling. Visualizing VCs by computer tomography is common practice. However, due to the low resolution of this method, it only allows the detection of large calcifications. The visualization of microcalcifications—the culprit behind plaque ruptures and acute vascular events—remains challenging, making it difficult to classify atherosclerotic calcifications. Consequently, the correct diagnosis of disease progression in the clinical setting appears difficult, limiting the application of drugs to prevent or treat VC. Currently, only *^18^*F-sodium fluoride-positron emission tomography can detect microcalcifications with high sensitivity, a method that received more attention for the diagnosis of VC over the past years [14]. 

Overall, VC is not solely responsible for vascular events since macro-calcifications may offer protection at the cost of vascular stiffness. In addition, clinicians lack proper diagnostic tools and drugs to identify and treat VC. This underlines the urgency to investigate further plaque heterogeneity and the cellular fate of the contributing cells.

### 1.2. The Role of Vascular Smooth Muscle Cells and Macrophages in Vascular Calcification

**Figure 1:**
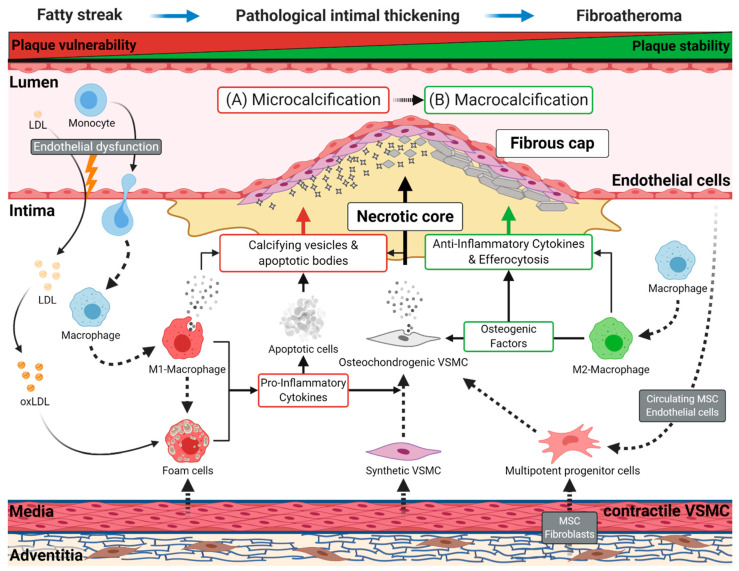
Schematic representation of atherogenesis and intimal vascular calcification. Endothelial dysfunction promotes retention of low-density lipoprotein (LDL) in the intima and the diapedesis of monocytes. Subsequently, retained LDL transforms to oxidized LDL (oxLDL), and monocytes migrating to the intima differentiate into macrophages. Later, these cells can switch to pro-inflammatory M1 or anti-inflammatory M2 macrophages. Resting macrophages and M1 macrophages can absorb oxLDL, which converts them into foam cells. These cells start forming the lipid-rich, necrotic core of the atherosclerotic plaque, which first occurs as a fatty streak in the vessel wall, the earliest sign of atherogenesis. During disease progression, vascular smooth muscle cells (VSMC) acquire a synthetic phenotype and migrate into the plaque contributing to foam cell formation and the stabilization of the fibrous cap. Foam cells and M1 macrophages induce a chronic, pro-inflammatory environment through the secretion of numerous cytokines. The resulting chronical inflammation and the subsequent consumption of oxLDL leads to the apoptosis of these lipid-laden cells and others cell types, causing the shedding of apoptotic bodies into the atherosclerotic plaque. (A) During early atherogenesis (e.g., pathological intimal thickening, fibroatheromas), apoptotic bodies originating from apoptotic cells, and calcifying vesicles, secreted by M1 macrophages, serve as the first nidus for microcalcifications, favoring unstable plaque formation. Subsequently, contractile VSMCs obtain a proliferative synthetic phenotype, leading to their migration into the intima to build the fibrous cap. The continuous inflammatory environment, induced by M1 macrophages and foam cells, initiates the osteochondrogenic transition of synthetic VSMC, contributing to micro- and macrocalcification. (B) In the further course of atherogenesis, M2 macrophages promote the progression of calcification through extracellular matrix deposition and by secreting various osteogenic factors, which may favor the development of sheet-like calcifications. Subsequently, M2 macrophages resolve inflammation by secreting anti-inflammatory cytokines and by removing apoptotic cells through efferocytosis. These mechanisms lead to regression and stabilization of the atherosclerotic plaque. Other vascular cells, originating from the adventitia (fibroblasts, pericytes, mesenchymal stem cells) or the endothelial cell layer, are potential sources for multipotent progenitor cells. The progenitor cells may contribute to the osteochondrogenic cell population in the plaque.

Early atherosclerotic disease results from a lesion known as a fatty streak, a visible collection of lipid-laden macrophages, called foam cells, in the vessel wall. This lesion develops to pathological intimal thickening, characterized by the migration of VSMCs from the vessel’s medial layer to the intima. Subsequently, a fibroatheroma arises, defined by a fibrous cap and a lipid-rich, necrotic core (Figure 1).

#### 1.2.1. The Osteochondrogenic Transition of VSMC during Vascular Calcification 

During pathological intimal thickening, VSMCs change from a contractile to a proliferative synthetic phenotype. This phenotype transition is characterized by the loss of contractile markers like calponin-1, actin alpha 2 (ACTA2, also known as α-smooth muscle actin), and by adopting a mesenchymal stem cell (MSC)-like plasticity [24,25]. The MSC-like plasticity indicates two key points: First, it seems to enable synthetic VSMC to acquire an osteochondrogenic phenotype to further accelerate plaque mineralization [25,26]. Second, this may indicate a signaling pathway activity comparable to MSC, in which osteogenesis and adipogenesis are balanced by competing transcription factors [27]. Endothelial cells and fibroblasts also can obtain this MSC-like plasticity, making them notable contributors to the osteochondrogenic cell population in the atherosclerotic lesion [26]. Furthermore, MSCs can migrate from the bloodstream into the atherosclerotic plaque, although their role for VC is not clear [28]. There is evidence that MSCs undergo an osteogenic transition when they are in contact with osteochondrogenic VSMC. This osteogenic transition might explain why advanced osteoid structures occur in advanced atherosclerotic plaques [29,30]. It further underlines the importance of the microenvironment for the fate of MSC, concluding that the pathological environment in atherosclerosis may drive MSC to become calcifying cells.

The main osteochondrogenic transcriptions factors SRY-box transcription factor 9 (SOX9) and runt-related transcription factor 2 (RUNX2, also known as CBFA1) are found in atherosclerotic plaques, being mainly expressed in osteochondrogenic VSMC [31,32,33,34]. These factors are essential during endochondral ossification, paving the way for MSC to differentiate to chondrocytes and, finally, to osteoblasts [11]. Similar to osteoblasts and chondrocytes, osteochondrogenic VSMC secrete extracellular vesicles loaded with calcium phosphate crystals and osteogenic factors like alkaline phosphatase (ALP) [35,36]. These vesicles may calcify on their own and further accelerate mineralization through the loaded osteogenic factors, making them an essential contributor to VC. In contrast, comparison of the transcriptome of osteochondrogenic VSMC- and MSC-derived osteoblasts revealed the heterogeneity of these cell types but also significant similarities in gene clusters responsible for the regulation of mineralization and the extracellular matrix (ECM) [37]. Compared to osteoblasts, osteochondrogenic VSMCs display enhanced apoptosis, reduced expression of osteogenic markers such as RUNX2, and decreased (ALP activity. The latter inhibits the endogenous calcification inhibitor pyrophosphate, hence, pathologically high ALP levels, as found in T2D or CKD, enable the deposition of hydroxyapatite crystals in the vessel wall [38]. Furthermore, calcifications close to osteochondrogenic VSMC in atherosclerotic plaques usually occur in small, discrete regions, while osteoblasts in bone tissue form large nodules associated with collagen deposition [39]. The different mineralization patterns of these two cell types may indicate that VSMCs do not undergo a complete transition to an osteoblast-like cell due to the atherosclerotic microenvironment. Instead, the trans-differentiation of VSMC may be halted during early atherogenesis, resembling a phenotype between chondrocyte and osteoblast. Consequently, VSMCs are essential for the induction of micro- and macrocalcifications in the vessel wall. However, these mechanisms are strongly influenced by the macrophage population’s inflammatory conditions in the lesion.

#### 1.2.2. The Role of Macrophages and Inflammation in Vascular Calcification

Macrophages act as an inflammatory modulator in atherosclerosis and are derived from monocytes migrating to the atherosclerotic plaque. Classically, the macrophage phenotypes were divided into M1 and M2 macrophages. Although this is a significant simplification since multiple macrophage subtypes are known, we will only refer to M1 and M2 macrophages in the following chapters, to ease the mechanistic understanding of this review [40].

As presented in Figure 1, inflammation is mainly mediated by M1 macrophages through the secretion of pro-inflammatory cytokines such as tumor necrosis factor alpha (TNFα), interleukin (IL) 1β or IL6 during early atherogenesis. The involved cytokines also start the osteochondrogenic program in VSMC, but persistent inflammation hinders further osteogenic maturation of the cells—a mechanism also observed during bone healing and inflammatory bone diseases [41,42]. The chronic inflammation keeps VSMC in an early osteochondrogenic state, while also increasing apoptosis. Both mechanisms trigger microcalcifications that, in turn, may further accelerate inflammation through the activation of macrophages [43]. On the other hand, M2 macrophages coordinate plaque regression by decreasing inflammation and secretion of osteogenic factors, leading to the maturation of osteochondrogenic VSMC and stabilizing the atherosclerotic plaque [41]. The M2 phenotype is present during early atherogenesis but may directly polarize to the pro-inflammatory M1 phenotype due to the chronically inflamed plaque microenvironment. Therefore, it was hypothesized that influencing the microenvironment may promote a switch back to M2 macrophages, which would support efferocytosis, ultimately leading to the clearance of dead cells and plaque regression [44]. As mentioned, M2 macrophages also have pro-osteogenic effects on VSMC, making the effect of this transition in the context of VC debatable [41]. Macrophages can also affect VC through the secretion of vesicles that contain Annexin 5 and S100A9, proteins that may aggregate with phosphatidylserine on the vesicle membrane, serving as a potential nucleation site for crystallization and, thus, may increase microcalcifications independent of osteochondrogenic differentiation of VSMC [45,46].

Various biochemical signaling networks, such as the bone morphogenetic protein (BMP)/transforming growth factor-beta (TGFβ) signaling and the receptor activator of nuclear factor-κB ligand (RANKL)/OPG/TNF–related apoptosis-inducing ligand (TRAIL) pathway are involved in the development of VC. Physiologically, the RANKL-OPG-TRIAL axis is essential for limiting bone loss due to osteoclasts, but dysregulation of this axis appears to play a detrimental role in the progression of VC [47]. Similar accounts for the BMP/TGFβ family, which regulates SOX9 and RUNX2 during endochondral ossification. The dysregulation of this pathway in VSMC is well known to play a role in their osteochondrogenic differentiation, and BMP-2 was found to induce WNT signaling activity. Notably, the WNT signaling pathway is essential for endochondral ossification by controlling the osteochondrogenic transition of MSC—a mechanism similar to the osteochondrogenic transition of VSMC during VC [31,32,33,34]. In MSC, the PPARγ signaling pathway inhibits endochondral ossification by interfering with the WNT signaling pathways, and comparable crosstalk in VSMC seems plausible [48]. The activation of PPARγ signaling demonstrated immunomodulatory properties in macrophages; thus, deciphering the interplay between PPARγ and WNT signaling in atherosclerosis may improve our understanding of VC [44].

## 2. The WNT Signaling Pathways

Nusse and Varmus described first parts of the WNT signaling network in 1982. Almost 40 years have passed, and WNT signaling has been shown to determine the fate of stem cells, making it essential for embryonal development and tissue homeostasis [49]. This signaling network is divided into three pathways; WNT/β-catenin-mediated signaling—commonly referred to as the canonical pathway, and the two β-catenin independent pathways, namely the planar cell polarity and WNT/Ca^2+^-mediated signaling, also known as the non-canonical pathways. The WNT ligand family consists of 19 lipid-modified, secreted glycoproteins, able to induce autocrine and paracrine signaling. In the cell, WNT/β-catenin signal transduction follows binding of a WNT ligand to a receptor of the frizzled (FZD) family and the lipoprotein receptor-related protein five or six (LRP5/6). Consequently, β-catenin accumulates in the nucleus and binds to transcription factors of the T-cell factor/lymphoid enhancer-binding (TCF/LEF) family, directing the transcription of the target gene [50]. Without WNT activation the intracellular β-catenin binds to the β-catenin destruction complex, consisting of glycogen synthase kinase 3-β (GSK3β), Axin, adenomatous polyposis coli (APC), and casein kinase-1 (CK1), and becomes phosphorylated. The ubiquitin–proteasome system then degrades phosphorylated β-catenin, hindering signal transduction. The β-catenin independent pathways, the planar cell polarity and the WNT/Ca^2+^ pathway, are transducing their signals in a different receptor context, involving FZD receptors and different co-receptors such as receptor tyrosine kinase-like orphan receptors one or two (ROR1/2) and related to receptor tyrosine kinase, while inhibiting WNT/β-catenin signaling. In addition, several regulatory molecules, namely the LRP5/6 inhibitors sclerostin, the dickkopf (DKK) protein family as well as the WNT ligand scavenging secreted frizzled receptor protein (sFRP) family, are regulating these molecular pathways. Aberrant WNT signal transduction plays an essential role in CVD, making this pathway a versatile subject to study in pathologies like VC as reviewed by our group [51].

### 2.1. The Role of WNT Signaling in the Initiation of Bone Development

Bone tissue develops either by intramembranous or endochondral ossification. During intramembranous ossification, MSCs directly differentiate towards osteoblasts to form the *calvaria* and clavicle bones. In contrast, endochondral ossification forms the remaining parts of the skeleton. In this process, chondrocytes develop from MSC and differentiate into osteoblasts. Numerous signaling pathways, including Hedgehog, Notch, BMP, as well as many others, control this developmental mechanism [27]. Especially, the WNT pathways play an inherent function in this developmental process by modulating the osteochondrogenic determinants SOX9 and RUNX2 in a stage-dependent manner [48]. 

The WNT signaling ligand WNT5a can start the chondrogenic program by inducing signal transmission through the WNT/Ca^2+^ pathway. This signal is mediated by Ca^2+^, which activates calcineurin (CaN) and, subsequently, the Ca^2+^/calmodulin-dependent protein kinase II (CaMKII). Afterwards, CaMKII dephosphorylates the nuclear factor of activated T-cells (NF-AT). This post-translational modification leads to the translocation of NF-AT to the cell nucleus, where it drives the transcription of the chondrogenic determinant SOX9 [52]. Consequently, this enables the transcription of SOX5 and SOX6, which further stimulates SOX9 transcription in a positive feedback loop, initiating chondrogenesis by modulating the gene expression of ECM proteins like collagen type II alpha (COL2A1) and aggrecan (ACAN) [53,54]. Concurrently, SOX9 facilitates the phosphorylation of β-catenin, leading to its degradation and hindering transcription of WNT/β-catenin target genes such as RUNX2. Accordingly, the degradation of β-catenin contributes to the disruption of chondrocyte maturation while also inhibiting the initiation of osteogenesis as a consequence [54,55]. On the other hand, SOX9 deficiency impairs limb development in mice by preventing RUNX2 expression in the skeleton, and the SOX-trio consisting of SOX5, 6 and 9, is essential to induce RUNX2 expression [56,57]. Therefore, functional SOX9 signaling is also vital to set the course for chondrocyte maturation and osteogenesis. 

During chondrocyte maturation, the stabilization of β-catenin plays an indispensable role. The stabilized β-catenin, together with TCF1, can bind to the promoter region of the osteogenic determinant RUNX2 to induce its transcription [58]. This mechanism initiates osteoblast differentiation by inducing protein expression essential for further osteoblast development, such as ALP, Osteocalcin (OCN), Osteopontin (OPN) and Osterix (OSX) [11,59]. 

The WNT signaling pathways are not only involved in endochondral ossification, but they also received considerable attention in connection with bone and cardiovascular diseases [51]. For instance, mutations of LRP5 can pathologically increase bone mass by inhibiting the binding of WNT inhibitors such as sclerostin or DKK1, whereas mutations of LRP6 are associated with CHD [60,61]. Consequently, it seems plausible to expect a role for the WNT signaling pathways in atherosclerosis and VC.

### 2.2. WNT Signaling in Vascular Calcification

As mentioned in Section 1.2, calcified atherosclerotic plaques, as well as synthetic and osteochondrogenic VSMC, display expression of SOX9 and RUNX2. Both transcription factors are regulated through WNT signaling and as a consequence, numerous downstream genes—important for ECM remodeling and calcification—such as COL2A1, ACAN, OCN, and ALP are found in calcified lesions [31,32,33,34].

#### 2.2.1. Non-β-Catenin-Mediated WNT Signaling and SOX9 in Vascular Calcification

The expression of WNT5a, an activator of the chondrogenic determinant SOX9, is found commonly in calcified atherosclerotic plaques [62,63,64]. In the radial arteries of patients with end-stage renal disease, this expression was shown to correlate with VC [65]. Similarly, the WNT5a concentration of serum was found to correlate with VC and CHD in obese patients with atherosclerosis [66]. This proposes that WNT5a contributes to VC, in turn, this also raises the question which cells are responsible for the secretion of WNT5a.

Recently published data revealed osteochondrogenic VSMC as a potential source for WNT5a, which seems plausible since WNT5a often is detected near calcifications [67]. However, the increase of WNT5a expression in VSMC was found in vitro and after the transition to the osteochondrogenic phenotype. Therefore, the initial pool of WNT5a, which potentially initiates the osteochondrogenic transition of VSMC, has to originate from elsewhere. 

Notably, WNT5a expression often locates near macrophage-rich regions, proposing macrophages as a potential source for WNT5a [62,63,64]. In macrophages, inflammatory factors like TNFα, IL6, or oxidized low-density lipoprotein (oxLDL) can induce WNT5a expression. Moreover, a positive feedback loop between IL6 and WNT5a exists in other diseases, which may indicate a similar mechanism in atherosclerosis [63,68]. The inflammatory factors present in the atherosclerotic plaque may drive macrophages to secrete WNT5a to promote the transition of VSMC to an osteochondrogenic phenotype. Equally important, the perivascular and the thoracic adipose tissue were described as potential sources for WNT5a, increasing the contributing cell pool further [66]. 

As mentioned in Section 1.2, MSCs may have a role in VC and, indeed, in vitro experiments showed that WNT5a/ROR2 signaling induces the osteochondrogenic transition of MSC when they are in direct contact with calcified VSMC, potentially accelerating VC [69]. However, MSCs that were indirectly co-cultivated with healthy VSMC during calcification, actually attenuated the osteochondrogenic transition of VSMC by interfering with WNT5a, ROR2 and β-catenin protein expression, indicating a protective mechanism under specific circumstances [70]. It is debatable, however, if there are comparable mechanisms in vivo, since no data are available.

#### 2.2.2. β-Catenin-Mediated WNT Signaling and RUNX2 in Vascular Calcification

A study on VC in rats with end-stage CKD did not show a significant increase in WNT5a but of the canonical WNT/β-catenin ligand WNT3a. Moreover, a correlation was found between the expression of β-catenin—the central signaling transducer of the WNT/β-catenin pathway—and VC [71]. These results align with numerous other studies that found an increased β-catenin activity or expression in osteochondrogenic VSMC and calcified atherosclerotic plaques [65,71,72,73]. The differential expression and activity of WNT5a and β-catenin indicate a switch from the non-β-catenin-mediated WNT signaling to the WNT/β-catenin pathway as occurring during endochondral ossification. The switch between these signaling pathways may demonstrate the continuous osteochondrogenic transition of VSMC and, thus, indicates the progression of atherosclerotic calcifications.

Calcified plaques display a high expression of RUNX2, which is essential for the full osteochondrogenic transition of VSMC [32,33]. Correspondingly, VSMC treated with high phosphate to simulate conditions observed in CKD, differentiate into osteochondrogenic VSMC while displaying high levels of RUNX2 [33,72,73,74]. Considering that RUNX2 expression relies on active WNT/β-catenin signaling in endochondral ossification, it seems plausible to expect a similar signal transduction in VC. For instance, Rats with CKD—a disease with a high prevalence for VC—display a positive correlation between active β-catenin and RUNX2 expression in the vasculature [71]. In VSMC, WNT/β-catenin ligands such as WNT3a, WNT7b, and WNT8b induce RUNX2 expression. Moreover, expression of transcriptional RUNX2 targets such as OCN, OSX, and ALP, is common in osteochondrogenic VSMC and calcified plaques [33,58,72,73,74]. The VSMC specific deletion of RUNX2 in LDLR^−/−^ mice abolishes the occurrence of AMC and AIC and the expression of OCN and ALP [33,75]. 

**Figure 2:**
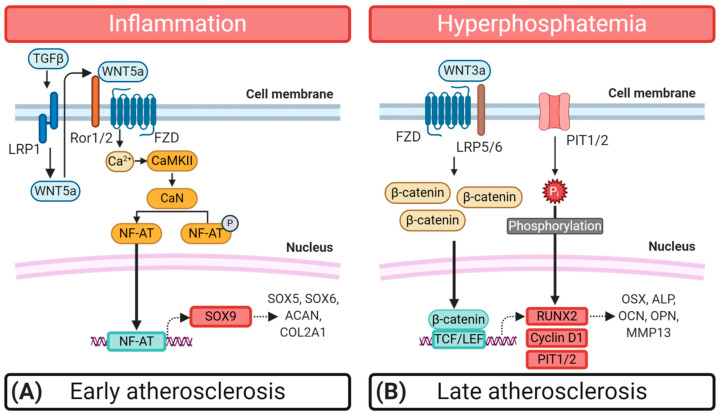
Schematic representation of the Wingless and Int-1 (WNT) signaling pathways and their role during the osteochondrogenic transition of synthetic vascular smooth muscle cells (VSMC). (**A**) Chronic inflammation stands at the beginning of early atherogenesis and initiates the β-catenin-independent WNT signal transmission, which sparks the osteochondrogenic differentiation of VSMC. Transforming growth factor β (TGFβ) binds to the low-density lipoprotein receptor 1 (LRP1), leading to the expression of WNT5a through an unknown mechanism in VSMC. WNT5a, mainly originates from surrounding macrophages and binds to frizzled receptors (FZD) and receptor tyrosine kinase-like orphan receptors 1 or 2 (ROR1/2). This activation induces the WNT/Ca^2+^ pathway involving Ca^2+^/calmodulin-dependent protein kinase II (CaMKII) and Calcineurin (CaN). Subsequently, CaN dephosphorylates the nuclear factor of activated T-cells (NF-AT), leading to the translocation of NF-AT to the nucleus where it initiates the transcription of the chondrogenic determinant SRY-box transcription factor 9 (SOX9), inducing the expression of extracellular matrix proteins like Aggrecan (ACAN), Collagen type II alpha (COL2A1), SOX5, and SOX6. (**B**) In late atherogenesis, conditions like hyperphosphatemia increase WNT/β-catenin signaling activity by inducing the expression of WNT/β-catenin ligands such as WNT3a. Binding of WNT3a to FZDs, co-activates lipoprotein receptor-related protein 5 or 6 (LRP5/6) inducing the recruitment of dishelved (DVL) protein to the receptor complex. Subsequently, DVL binds the β-catenin destruction complex, containing glycogen synthase kinase 3β (GSK3β), axin, adenomatous polyposis coli (APC) and casein kinase-1 (CK-1). This leads to an accumulation of β-catenin in the cytoplasm, followed by its translocation to the nucleus where it forms a complex with T-cell factor/lymphoid enhancer factor (TCF/LEF) to induce transcription of the osteogenic determinant runt-related transcription factor 2 (RUNX2) and the PPARγ inhibitor Cyclin D1. RUNX2 controls the transcription of osteogenic factors such as Osteocalcin (OCN), Osterix (OSX), Osteopontin (OPN), matrix metalloprotease 13 (MMP13), and alkaline phosphatase (ALP).

In essence, the aberrant WNT5a/SOX9 signal transmission in atherosclerosis seems to set the stage to initiate atherosclerotic calcification (Figure 2A). Later on, a switch to WNT/β-catenin signaling occurs, which is required for RUNX2 expression, to ultimately drive the osteochondrogenic transition of VSMC in VC (Figure 2B). 

### 2.3. The Role of WNT Inhibitors

The role of the DKK protein and secreted frizzled-related protein (sFRP) family in bone development, atherosclerosis and VC has been discussed in detail recently [76,77,78]. For this reason, we will further address the relationship between WNT inhibitors and VC in the following chapter.

#### 2.3.1. The Dickkopf Protein Family

DKK1 was the first discovered DKK protein and, like DKK2 and DKK4, is known to antagonize WNT/β-catenin signaling by acting together with Kremen (Kre) co-receptors to direct the endocytosis of LRP5/6, inhibiting further signal transduction [51]. Several studies correlated elevated DKK1 serum or plasma concentration, with the prevalence of CVD and particularly vascular events, indicating a specific role in atherosclerosis [79,80,81,82]. 

In the vasculature, platelets and endothelial cells are sources of DKK1 [79,80]. Pro-atherosclerotic factors like shear stress or oxLDL are able to induce DKK1 expression in endothelial cells. Additionally, platelet activation through thrombin increases DKK1 secretion [79,83,84]. This excessive DKK1 secretion may contribute to inflammation, monocyte adhesion, and apoptosis of endothelial cells, ultimately, promoting endothelial dysfunction—the initiating step to atherosclerosis. Furthermore, DKK1 regulates the de-differentiation of endothelial cells to MSC, a mechanism commonly found in atherosclerosis [85,86]. The endothelial-to-mesenchyme transition could contribute to the amount of MSCs in the atherosclerotic plaque. In the presence of osteochondrogenic VSMC, these MSC tend to undergo an osteogenic transition, which likely promotes VC [69]. In contrast, an inverse correlation between low serum levels of DKK1 and VC was found in several studies, while in vitro DKK1 also attenuates calcium deposition and expression of RUNX2 in osteochondrogenic VSMC [72,74,87,88]. Consequently, DKK1 may limit the osteochondrogenic transition of VSMC, which could prolong the occurrence of instable atherosclerotic plaques by hindering the build-up of stabilizing, sheet-like calcifications during late atherogenesis. This and the negative effects on endothelial function might explain the strong association between DKK1 and CVD. 

The knowledge about other DKK proteins and their function in atherosclerosis and VC remains limited. In osteoblasts, DKK2 may activate the WNT/β-catenin cascade and modulate osteogenic signaling, whereas DKK4 seems to suppress osteogenic lineage determination in bone marrow-derived MSC [89,90,91,92]. Henceforth, it is questionable if both factors also affect the synthetic VSMC—osteochondrogenic transition. 

In contrast to the other DKK proteins, DKK3 performs its action through binding of Krm1 or Krm2 and not by binding LRP6, however, the signaling pathways influenced by DKK3 are complex and not fully understood [76,93]. In atherosclerosis, DKK3 may enhance plaque stability by inducing the transition of fibroblast to VSMC and increasing ECM deposition, both essential factors for VC [94]. This may suggest a role for DKK3 in promoting VC, although this hypothesis remains to be studied. 

#### 2.3.2. The Secreted Frizzled-Related Protein Family

The sFRP family is well-known to antagonize both β-catenin dependent and independent WNT signaling by scavenging WNT ligands and by interacting with FZD receptors to inhibit the binding of other ligands. Nevertheless, the sFRP protein family may also act as WNT ligand transporters, giving them a putative function in WNT signal transduction. Despite the broad expression of these proteins in aortic VSMC, their role in controlling WNT signaling during VC appears unclear [95]. 

In fibroblasts, sFRP1 controls chondrogenic fate by attenuating SOX9, RUNX2 and COL2A1, which was associated with decreased WNT/β-catenin signaling [96]. Mice without sFRP1 expression exhibit an increased RUNX2 and OCN expression as well as increased bone mass, indicating that sFRP1 loss leads to a faster chondrocyte maturation and, consequently, earlier induction of osteogenesis [58]. Similarly, sFRP2 reduces the osteochondrogenic transition of VSMC, by scavenging WNT5a, which attenuated SOX9 transcription [64]. Consequently, both factors are linked to VC. Besides, sFRP1 and sFRP2 suppress or activate WNT3a-mediated WNT/β-catenin signaling depending on the ligand- and receptor combination as well as the presence of WNT3a. For instance, increasing the availability of sFRP1, sFRP2, and WNT3a abolishes WNT/β-catenin signaling when FZD5 is not expressed in the target cell or tissue [97]. In obese patients with atherosclerosis, however, the expression of FZD5 rises, potentially weakening the function of sFRP1 and sFRP2 as WNT/β-catenin inhibitors [66]. As a consequence, their potential to attenuate VC in atherosclerosis may be impaired. 

Various clinical studies have found lower sFRP5 serum levels in obese, T2D, and CHD patients—all high-risk groups for VC—suggesting a critical function for sFRP5 in the healthy vasculature [66,98,99]. Moreover, increased WNT5a levels in obese patients were found, and some author suggested a link between sFRP5 and WNT5a in atherosclerosis [66,100]. It was further demonstrated that sFRP5 might inhibit WNT5a signaling, showing its potential to inhibit VC. In contrast, a recent publication found that WNT3a/β-catenin signaling decreases in VSMC when treated with sFRP5, consequently attenuating RUNX2 expression. Thus, the osteochondrogenic de-differentiation of VSMC was impaired, and a potential role for sFRP5 in inhibiting VC by scavenging WNT3a was suggested [101]. However, this study could not confirm a direct interaction between Wnt3a and sFRP5. Considering the function of WNT5a in endochondral ossification, treatment of VSMC might has caused inhibition of WNT5a, which, consequently, would suppress the osteochondrogenic differentiation of VSMC. 

Overall, since they are able to inhibit WNT5a/SOX9 as well as WNT/β-catenin signal transduction, sFRPs may turn out as the first line of defense in VC. However, our understanding is still limited, and further studies are required to explain how the distribution of sFRP, FZD and WNT molecules in different cell types influence VC.

## 3. Peroxisome Proliferator-Activated Receptor-γ Signal Transduction

PPARγ belongs to a family of nuclear receptors that includes the subtypes PPARβ/δ and PPARα. This receptor family coordinates a wide range of metabolic mechanisms like insulin sensitivity, glucose homeostasis and lipogenesis. PPARγ, in particular, is the main determinant that regulates adipogenesis.

Naturally occurring polyunsaturated fatty acids like eicosapentaenoic acid (EPA) and synthetic ligands such as thiazolidinedione’s (TZD) can directly activate PPARγ. Subsequently, PPARγ forms a heterodimer with the retinoid X receptor (RXR), and the resulting receptor complex binds to the PPAR-response element (PPRE) in the promoter region of numerous target genes, ultimately inducing their transcription. In contrast, target genes with PPRE binding sites can also be suppressed by the PPARγ–RXR complex as long as no ligand is available. Additionally, ligand-bound PPARγ binds, without RXR, to specific target sites to repress target genes [102,103].

### 3.1. PPARγ Signaling in Atherosclerosis

VSMC in the vasculature and macrophages express PPARγ, indicating a role for PPARγ in vascular function, inflammation, and, consequently in vascular disease [104,105]. Indeed, suppression or deficiency of PPARγ is known to accelerate atherosclerotic lesion formation in different animal models. For instance, dominant-negative mutations of PPARγ in the aorta and VSMC lead to vascular dysfunction and hypertrophy in mice [104,105]. In atherosclerotic plaques of the *arteria carotis*, RXRα and PPARγ expression is reduced in the VSMC and macrophage cell populations [106]. The treatment with TZDs, on the other hand, reduces atherosclerotic plaque volume in ApoE^−/−^ mice, New Zealand rabbits, as well as in CAD and T2D patients [107,108,109,110]. Even though these animal and clinical models differ significantly from each other, all studies concluded that the atherosclerotic plaque regression was due to PPARγ activation. Consequently, PPARγ seems essential for the healthy vasculature and PPARγ suppression may favor atherogenesis. The positive effects of PPARγ in atherosclerosis may occur due to its anti-inflammatory effects in the vasculature. Since inflammation and VC are strongly entangled, it stands to reason if the anti-inflammatory effects of PPARγ also attenuate VC by inhibiting the osteochondrogenic transition of VSMC.

In calcified radial arteries from CKD patients and calcified aortas from CKD mice, PPARγ expression decreases while osteochondrogenic VSMC cultured in high-phosphate or -glucose conditions present a similar outcome [111,112]. Concluding, that hyperphosphatemia and hyperglycemia, conditions commonly found in CKD and T2D, may attenuate PPARγ signal transduction in vivo, favoring VC. 

### 3.2. PPARγ and the Vascular Calcification—Inflammation Axis

High levels of pro-inflammatory cytokines such as TNFα and IL6 are frequently observed in CKD, T2D, and CAD patients, leading to chronic low-grade inflammation of the vasculature while also correlating with cardiovascular events [113,114,115,116]. Aforementioned in chapter 1.2, TNFα, IL1β, and IL6 are secreted by M1 macrophages. These cytokines favor the osteochondrogenic transition of VSMC and, consequently, the development of VC in mice [117,118,119,120]. Upon stimulation with a pro-inflammatory stimulus like Lipopolysaccharide, PPARγ deficiency increases TNFα, IL1β, and IL6 secretion in macrophages, an effect that indicates the transition to an M1 like phenotype [121]. On the other hand, PPARγ activation primes monocytes to undergo the transition to anti-inflammatory M2 macrophages, which are associated with plaque regression in CAD [44,122,123,124]. As a consequence, this may abolish the development of inflammation-mediated microcalcifications, hence, ensuring plaque stability. These evidences underline the importance of PPARγ in shaping the macrophage phenotype, making it a potential target to treat VC. 

TNFα is essential for the inflammatory response during atherogenesis, and various pro-atherosclerotic factors induce TNFα expression in macrophages, including TGFβ, interferon-γ, as well as autocrine stimulation through TNFα itself [125]. In contrast to IL6, TNFα serum concentration does not correlate directly with CAC in CKD patients but both cytokines correlate positively, indicating interdependency [116]. The pro-osteogenic effects of TNFα seem to occur through the induction of IL6 via the c-FOS/activator protein-1/nuclear factor kappa-light-chain-enhancer of activated B cells (NF-κB) pathway in VSMC, which in turn accelerates the expression of RUNX2, ALP and other osteogenic factors [117]. However, early-stage T2D patients treated with TZD display a reduced TNFα and IL6 serum concentration, and monocytes react similarly to this treatment [126,127]. In conclusion, the mentioned data confirms the influence of inflammatory cytokines and the role of PPARγ suppression in VC by modifying the immune response of macrophages. 

### 3.3. Interplay of PPARγ Signaling with Other Signaling Pathways in Vascular Calcification

The single transmembrane protein α-Klotho is a phosphaturic protein essential for regulating mineral homeostasis and vitamin D metabolism. It is mainly expressed in the kidney and functions a co-receptor for fibroblast growth factor (FGF) 23 to promote binding to its target receptors. The membrane-bound α-Klotho can be cleaved by secretases, leading to its secretion in blood, where it fulfills regulatory functions. A loss of α-Klotho in mice is known to provoke a premature aging syndrome, which leads to the appearance of atherosclerosis, VC, and other age-related pathologies [128]. 

The chronically high concentration of pro-inflammatory cytokines like IL6 and TNFα found in atherosclerosis and T2D attenuates renal and vascular Klotho expression by increasing FGF23 concentration through the NF-κB pathway [128,129,130]. This finding links chronic low-grade inflammation to α-Klotho deficiency, highlighting its relevance for VC.

During CKD progression, secreted α-Klotho concentration decreases, ultimately leading to hyperphosphatemia and VC [128,131,132]. This decrease of α-Klotho is due to a pathological increase in FGF23, parathyroid hormone, and low vitamin D levels, a deteriorated serum profile that frequently occurs as CKD progresses [128]. Similar effects are observed when VSMC were calcified under high-phosphate conditions, leading to α-Klotho deficiency accompanied by an increase of inorganic phosphate transporter (PIT) 1 and 2 expression—transporter proteins essential for P_i_ influx in VSMC—indicating an interdependency of VC and hyperphosphatemia [111,132,133]. However, mice fed a high-fat diet (HFD) display no change in phosphate serum concentration but increased TNFα and FGF23 [134]. Similarly, in rats fed HFD, phosphate restriction did not improve the FGF23 or secreted α-Klotho serum profile [135]. These observations were further evidenced in HFD fed rabbits, which displayed a decreased α-Klotho expression accompanied by an upregulation of calcification markers like OPG and OPN, as well as an imbalance of different transporter proteins such as PIT1 and enzymes responsible for the regulation of a healthy inorganic phosphate and pyrophosphate ratio [136]. Consequently, the serum phosphate concentration alone, probably, does not predict VC, but rather the ratio of inorganic phosphate and the calcification inhibitor pyrophosphate. Overexpression of α-Klotho, on the other hand, abolishes the osteochondrogenic transition of VSMC by downregulating PIT1 and PIT2, effects that were also achieved with PPARγ agonists achieved in CKD mice [111,132,137].

The acetylation of PPARγ is essential to regulate the transcription of various target genes. However, histone deacetylases (HDAC) like HDAC3 are upregulated in mice with CKD, leading to the suppression of PPARγ activity through de-acetylation, which may explain the α-Klotho deficiency in CKD [138]. Consequently, PPARγ activation during atherogenesis may upregulate α-Klotho to interfere with the osteochondrogenic changes of VSMC.

Activation of PPARγ also suppresses the renin angiotensin aldosterone system (RAAS) [128,139]. The RAAS is associated with hypertension, vascular dysfunction, and an α-Klotho inhibitor. A recently published study suggested crosstalk between PPARγ, RAS, and Klotho in a CKD animal model and MDCK cells [140]. Still, the impact of the RAAS on VC is unclear, but current advances show that mice, over-expressing the angiotensin type 2 receptor in VSMC, display a higher PPARγ activity and were less susceptible to VC. Additionally, the PPARγ antagonist GW9662 attenuated the effects of angiotensin type 2 receptor overexpression and PPARγ activity in rats and VSMC [141,142]. Despite the clear connection between Klotho, PPARγ, and VC, further research is needed to understand the involvement of the RAAS.

## 4. The Interplay between PPARγ and WNT Signaling in Vascular Calcification

**Figure A1:**
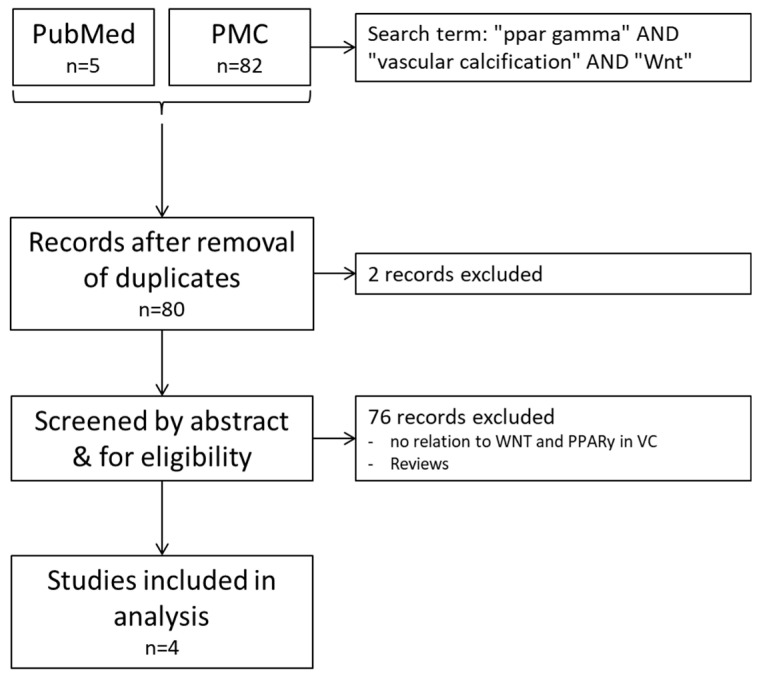
Flow chart to illustrate the systematic search protocol to identify relevant literature in the PubMed and PMC databases.

The following free and Mesh search terms were used to identify peer reviewed articles in English through the electronic databases PubMed and PMC: “ppar gamma” AND “vascular calcification” AND “Wnt”. Reviews, Editorials and conference abstracts were excluded. In addition, all studies were excluded that did not show any relations to WNT and PPARγ in the context of VC. Searches were performed on 10 July 2020 (Figure A1) and in total four studies (Woldt et al.; Zhou et al.; Gao et al.; and Saito et al.) were included.

The suppression of PPARγ, is fundamental for the osteogenic lineage determination of MSC [27,48]. However, the differentiation from MSC to osteoblasts requires WNT/β-catenin signaling activity, mediated by ligands like WNT10b [143]. The higher WNT/β-catenin signaling activity attenuates PPARγ signal transduction via β-catenin stabilization and transcriptional targets like cyclin D1 [143,144,145,146]. This relationship has received growing attention over the past years, especially in MSC, and some authors already reported a differential expression of the WNT/β-catenin pathway and PPARγ in the atherosclerotic plaque [48,147]. Literature about the crosstalk between β-catenin independent WNT signaling and PPARγ, however, remained scarce and was covered by just a few studies. In VC, it remains unclear if and how these pathways are interconnected, questioning if VC partly result from a dysfunctional interplay between WNT and PPARγ signal transduction.

### 4.1. PPARγ Signaling Crosstalk with the Non-β-Catenin-Mediated WNT Signaling Pathway

A PPARγ and β-catenin independent WNT signaling interplay was first evidenced by Woldt et al. In their study, PPARγ deficiency in VSMC of LDLR^−/−^ mice increased VC, which was accompanied by an increased SOX9 and RUNX2 expression [64]. In accordance with studies on chondrogenic precursor cells, the study demonstrated that WNT5a induces SOX9 expression, initiating the stage-dependent, osteochondrogenic differentiation of VSMC [52,64]. The deletion or suppression of LRP1—a receptor essential for early chondrogenesis—was associated with the absence of calcified lesions and a decreased WNT5a expression in the vessel wall in mice, indicating a function for LRP1 in VC by upregulating WNT5a expression [64,148]. This LRP1-WNT5a link was also evidenced in fibroblasts, where TGFβ treatment—a factor known to originate from the injured vessel wall—of LRP1-deficient fibroblasts did not induce WNT5a transcription and provoked a considerably lower protein induction than found in control fibroblasts [149,150,151]. Notably, TGFβ is also a well-known ligand for LRP1, where it plays a role in fibroblast activation and ECM synthesis [152]. Further, TGFβ promotes osteogenesis and migration of MSC, while also inducing osteochondrogenic effects on VSMC. Therefore, a hypothetical TGFβ/LRP1/WNT5a/NF-AT/SOX9 signaling cascade could set the stage for VSMC-mediated VC (Figure 2A) [28,153]. 

This cascade may be inhibited by the activation of PPARγ via the upregulation of the WNT5a scavenger’s sFRP2 and sFRP5 [64,154]. Current advances demonstrated that sFRP5 seems to attenuate the osteochondrogenic transition of VSMC through the inhibition of WNT/β-catenin signaling (as discussed in Section 2.3) [95,101]. However, sFRP5 inhibits WNT5a signal transduction in adipose tissue and macrophages, hindering inflammation and macrophage activation [155,156]. Consequently, sFRP5 possibly also inhibits WNT5a in osteochondrogenic VSMC. However, calcified VSMC displayed a low WNT5a and sFRP5 expression [95]. This appears contradictive at first, but indeed is comparable to endochondral ossification, during which WNT5a initiates the chondrogenic transition, but must be inhibited to complete the remaining osteogenic program. The demonstration for this sequential mechanism, however, is missing in the study reviewed. This highlights the obligation to perform longitudinal studies to investigate the time-dependent character of molecular signaling events in VC. Beyond the local impact of sFRPs mediated via PPARγ activation on VSMC, a recent study has evidenced the ability of sFRP5 to directly interact with LRP1 in macrophages in pro-inflammatory conditions. Thereby, it induces its proteasomal degradation, as suggested by the authors [157]. This may result in increased availability of WNT5a originating from macrophages, which might promote the osteochondrogenic transition of VSMC in the atherosclerotic plaque.

### 4.2. The Effects of PPARγ Signaling on β-Catenin-Mediated WNT Signaling

**Figure 3:**
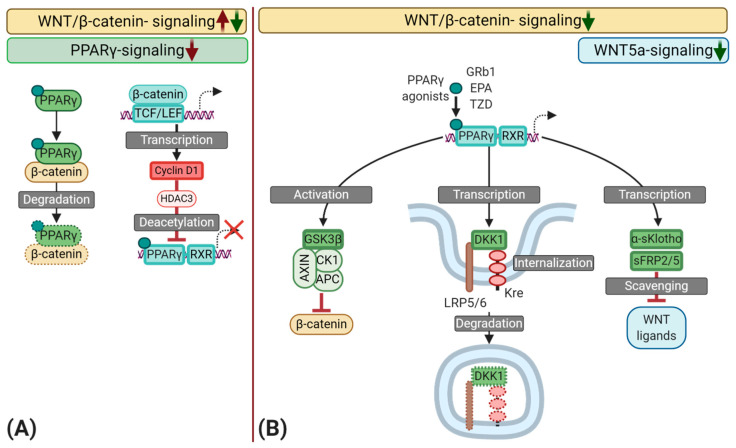
Schematic representation of the interplay between peroxisome proliferator-activated receptor γ (PPARγ) and the Wingless and Int-1 (WNT) signaling pathways during vascular calcification (VC) with a focus on the osteochondrogenic transition of synthetic vascular smooth muscle cells (VSMC). (**A**) Several mechanisms may inhibit PPARγ signaling during atherogenesis. First, ligand-bound PPARγ may sequester β-catenin, leading to the proteasomal degradation of this complex. As a result, a high activation of the WNT/β-catenin pathway could reduce PPARγ availability and vice versa. Second, WNT/β-catenin signaling induces the transcription of Cyclin D1, which promotes the recruitment of Histone deacetylase 3 (HDAC3) to PPARγ, inducing deacetylation, consequently, reducing the transcriptional activity of PPARγ. (**B**) Various naturally occurring compounds such as polyunsaturated fatty acids like eicosapentaenoic acid (EPA) and the ginsenoside Rb1 (GRb1), as well as synthetic compounds like Thiazolidines (TZD), can directly activate PPARγ. Upon activation, PPARγ interacts with retinoid-x-receptors (RXR) to induce the transcription of target genes. PPARγ may dimmish WNT/β-catenin signaling through the activation of glycogen synthase kinase-three-β (GSK3β)—an essential part of the β-catenin destruction complex. Moreover, PPARγ induces the transcription of WNT scavengers like secreted frizzled-related protein 2 and 5 (sFRP2/5), as well as secreted α-Klotho (α-sKlotho), which results in an inhibition of SOX9 and RUNX2 by scavenging WNT ligands such as WNT5a and WNT3a. The binding of dickkopf-1 (DKK1), another transcriptional target of PPARγ, to lipoprotein receptor-related protein 5 or 6 (LRP5/6) leads to a complex formation with the Kremen receptor (Kre), followed by its internalization and degradation, ultimately inhibiting Wnt/β-catenin signal transduction.

PPARγ may also interfere with the pro-osteogenic effects of WNT/β-catenin signaling (Figure 3). The activation of PPARγ inhibits the osteogenic transition of MSC by interfering with RUNX2 expression and collagen deposition and comparable results were reported in osteochondrogenic VSMC of the rat [158,159]. Gao et al. demonstrated that the calcium deposition as well as RUNX2, β-catenin, and Cyclin-D1 expression was decreased after treatment with Pioglitazone [160]. Similarly, Zhou et al. obtained comparable results with the PPARγ agonist ginsenoside Rb1 (GRb1) in osteochondrogenic VSMC [161,162,163]. In the control group, a decreased expression of the contractile markers calponin-1 and ACTA2 as well as an increased expression of RUNX2, accompanied by high β-catenin activity was observed. This indicates the phenotypic transition of the VSMC to an osteochondrogenic phenotype. However, treatment with GRb1 attenuated the osteochondrogenic transition and increased PPARγ expression, whereas a PPARγ antagonist reversed these effects. The authors concluded that PPARγ mediated these anti-osteogenic effects, demonstrating a PPARγ and WNT/β-catenin crosstalk of relevance for VC [163]. Other PPARγ agonists, such as the omega-3-fatty acid EPA—as described by Saito et al. and others—are also known to reduce VC in different rodent models [164,165,166,167]. 

Aforementioned in Section 2.3, α-Klotho is a transcriptional target of PPARγ. Moreover, Klotho^−/−^ mice display a high expression of β-catenin in the aorta [165]. These findings may indicate that Klotho inhibits the WNT/β-catenin pathway in VC and, indeed, secreted α-Klotho sequesters WNT/β-catenin ligands; consequently, inhibiting WNT/β-catenin signal transduction [168,169]. In human VSMC, EPA abolishes WNT3a-induced osteochondrogenic changes by decreasing RUNX2 and increasing PPARγ expression [165]. Hence, EPA may attenuate VC by acting on PPARγ to inhibit WNT/β-catenin activity through the upregulation of Klotho. However, α-Klotho also attenuates PIT1 and PIT2 gene expression—Phosphate transporters, which are essential for the osteochondrogenic transition of VSMC [132]. Consequently, the effects found in this study could be partly initiated through a downregulation of PIT1 and 2.

Besides the transcription of α-Klotho, PPARγ may induce activation of the WNT/β-catenin inhibitor GSK3β, and the transcription of DKK1 [160,170,171]. The latter observations were obtained in adipocytes, making a direct translation to VSMC difficult. However, the used cell types originate from MSC, which could hint to a similar conserved pathway in VSMC. Moreover, GSK3β activity in the β-catenin destruction complex does not depend on its serine or tyrosine phosphorylation, still this measurement was used in this study to determine GSK3β-activity. Consequently, it is not clear if these effects were related to PPARγ [160,172]. 

Beyond these previous observations, ligand-bound PPARγ may sequester β-catenin through its TCF/LEF binding domain and a catenin binding domain, a process that may occur GSK3β-mediated and -independent as well, leading to the proteasomal degradation of this complex via ubiquitination [173,174,175]. Given the delicate balance between WNT and PPARγ in VC, this could be a double-edged sword, since a high activation of the WNT/β-catenin pathway, as found in atherosclerosis, could reduce PPARγ availability, shifting the balance towards a pathologically high β-catenin concentration. Furthermore, oncogenic forms of β-catenin show resistance towards the β-catenin destruction complex and inhibit binding of PPARγ, questioning the presence of similar mutations in atherosclerotic lesions [174].

The WNT/β-catenin signaling target Cyclin D1 initiates the recruitment of HDAC3 to PPARγ, leading to the de-acetylation of PPARγ, ultimately suppressing target gene transcription [144]. Notably, HDAC3 expression is increased by inflammatory factors like TNFα and inhibiting HDAC1 and -3 with MS-275 attenuates calcification of valvular interstitial cells, accompanied by a reduced WNT/β-catenin signaling activity and RUNX2 expression [176,177]. However, the effect of this HDAC Inhibitor is not specific and inhibits other HDACs like HDAC1, which may inhibit VC in specific settings [178]. Regardless, it stays questionable if the described mechanisms are similar in VSMC. 

## 5. Conclusions

In this review, we described how the sequential activation of WNT5a-signaling, the WNT/β-catenin pathway and the dysregulation of PPARγ during atherogenesis, is involved in VC by guiding the osteochondrogenic transition of VSMCs. Plaque topography, disease etiology, as well as cell heterogeneity and the complexity of the underlying mechanisms make it essential to investigate these fundamental molecular pathways further. The resulting insights may clarify the relationship of macrophages and VSMC during VC, by identifying key factors relevant for the induction of WNT signaling in atherosclerotic plaques. Besides some new, experimental, pharmaceuticals like the RANKL inhibitor Denosumab, no commercial drug affects the main cause of VC, namely the osteochondrogenic differentiation of VSMC. Therefore, already registered PPARγ agonists may deserve more attention as potential VC inhibitors for patients at the onset of atherosclerosis due to their anti-inflammatory and cell fate modulating properties.

## Abbreviations

ACAN	Aggrecan
ACTA2	α-smooth muscle actin
AIC	arterial intimal calcification
ALP	Alkaline phosphatase
AMC	arterial medial calcification
APC	Adenomatous polyposis coli
BMP	Bone morphogenetic protein
CAC	Coronary artery calcification
CaN	Calcineurin
CHD	Coronary heart disease
CK1	Casein Kinase-1
CKD	Chronic kidney disease
COL2A1	collagen type II alpha
CVD	Cardiovascular disease
DKK	Dickkopf
ECM	Extracellular matrix
FGF	Fibroblast growth factor
FZD	Frizzled receptor
GRb1	Ginsenoside Rb1
GSK3β	Glycogen synthase kinase 3-β
HDAC	Histone deacetylase
HFD	High-fat diet
IL	Interleukin
Kre	Kremen
LRP5/6	Lipoprotein receptor-related protein 5/6
MSC	Mesenchymal stem cells
MMP13	Matrix metalloprotease 13
NF-AT	nuclear factor of activated T-cells
NF-κB	kappa-light-chain-enhancer of activated B cells
OCN	Osteocalcin
OPG	Osteoprotegerin
OPN	Osteopontin
OSX	Osterix
oxLDL	oxidized low density lipoprotein
PIT	inorganic phosphate transporter
PPARγ	Peroxisome proliferator-activated receptor γ
PPRE	PPAR response element
RANKL	receptor activator of nuclear factor-κB ligand
RUNX2	Runt-related transcription factor 2
RAAS	Renin angiotensin aldosterone system
RXR	Retinoid X receptor
sFRP	secreted frizzled receptor protein
SOX9	SRY-box transcription factor 9
T1D	Type 1 Diabetes
T2D	Type 2 Diabetes
TCF/LEF	T-cell factor/lymphoid enhancer-binding factor
TGFβ	Transforming growth factor β
TNFα	Tumor necrosis factor α
TRAIL	TNF–related apoptosis-inducing ligand
VC	Vascular calcification
VSMC	Vascular smooth muscle cells
WNT	Wingless/Int-1
